# Supplementary material for: Correlation between superconductivity and bond angle of CrAs chain in non-centrosymmetric compounds A2Cr3As3 (A = K, Rb)
Source: Sci Rep. 2016 Nov 25;6:37878. doi: 10.1038/srep37878 (PMC5122944; doi:10.1038/srep37878)
Supplement: Supplementary Material [file srep37878-s1.pdf]

Supplementary Material for "Correlation between superconductivity and bond angle of CrAs chain in non-centrosymmetric compounds $A_2Cr_3As_3$ (A=K, Rb)"

Zhe Wang[1]*, Wei Yi[1]*, Qi Wu[1], Vladimir A. Sidorov[2], Jinke Bao[3], Zhangtu Tang[3], Jing Guo[1], Yazhou Zhou[1], Shan Zhang[1], Hang Li[1], Youguo Shi[1], Xianxin Wu[1], Ling Zhang[4], Ke Yang[4], Aiguo Li[4], Guanghan Cao[3], Jiangping Hu[1,5], Liling Sun[1,5]† & Zhongxian Zhao[1.5]

[1]*Institute of Physics and Beijing National Laboratory for Condensed Matter Physics, Chinese Academy of Sciences, Beijing 100190, China*

[2]*Institute for High Pressure Physics, Russian Academy of Sciences, 142190 Troitsk, Moscow, Russia*

[3] *Department of Physics, Zhejiang University, Hangzhou 310027, China*

[4]*Shanghai Synchrotron Radiation Facilities, Shanghai Institute of Applied Physics, Chinese Academy of Sciences, Shanghai 201204, China*

[5]*Collaborative Innovation Center of Quantum Matter, Beijing, 100190, China*

† Correspondence and requests for materials should be addressed to:

L.L.S. (llsun@iphy.ac.cn)

*These authors are contributed equally.

Crystal structure refinements of the high pressure XRD data and estimation of electron density distributions for the pressurized sample

For the crystal structure refinements of the high pressure XRD data (Fig.S1 and Fig.S2) with the Rietveld method [1], the program RIETAN-FP was employed [2-5], and the ambient-pressure lattice parameters of the host sample were used as the initial data. In the refinements, the occupancy for all the positions of atoms was taken as 100%. The electron density distributions (EDD) were determined by maximum entropy method (MEM) based on our XRD data with Dysnomia program [6]. In the EDD calculations, the unit cell was divided into 132×132×64 pixels along the three axial directions of the unit cell. The crystal structures and the EDD were visualized using the software package VEST [7].

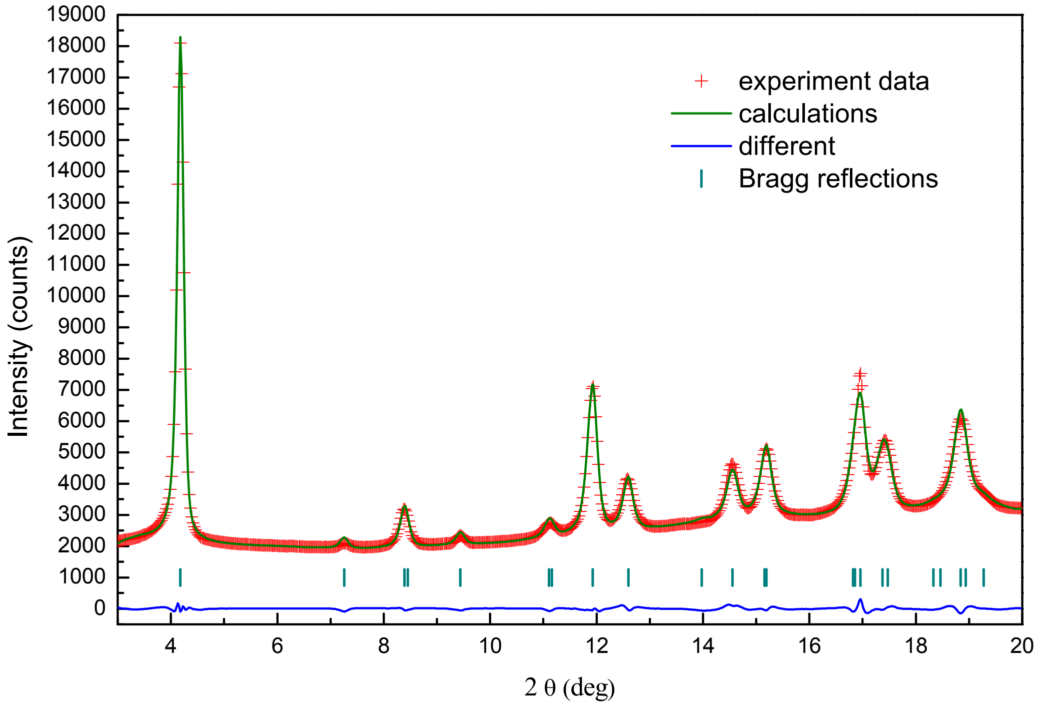

Figure S1 Synchrotron X-ray powder diffraction patterns (measured with λ = 0.61992 Å) for $K_2Cr_3As_3$ at 1.35 GPa.

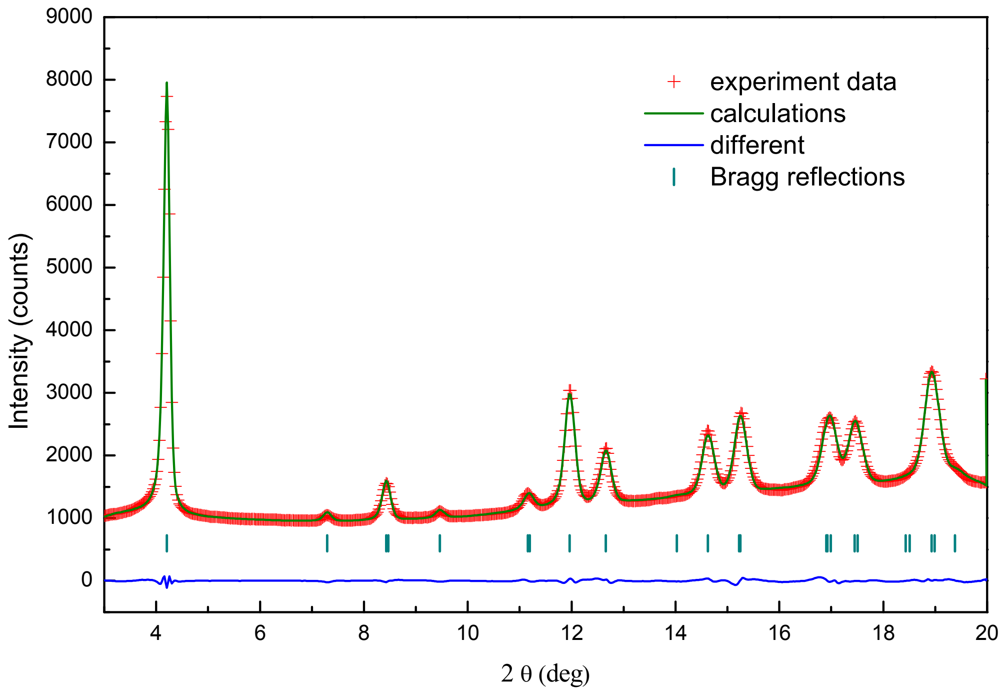

Figure S2 Synchrotron X-ray powder diffraction patterns (measured with λ = 0.61992 Å) for $K_2Cr_3As_3$ at 3.39 GPa.

References

[1] H. M. Rietveld, J. Appl. Crystallogr. **2**, 65 (1969).

[2] F. Izumi and K. Momma, Solid State Phenom. **130**, 15 (2007).

[3] F. Izumi, S. Kumazawa, T. Ikeda, W. Z. Hu, A. Yamamoto, and K. Oikawa, Mater. Sci. Forum **378-381**, 59 (2001).

[4] F. Izumi and T. Ikeda, Mater. Sci. Forum **321-324**, 198 (2000).

[5] F. Izumi and R. A.Young, *The Rietveld Method*, (Oxford University Press, Oxford, 1995).

[6] K. Momma, T. Ikeda, A. A. Belik, and F. Izumi, Powder Diffr. **28**, 184 (2013).

[7] K. Momma and F. Izumi, J. Appl. Crystallogr. **44**, 1272 (2011).
